# Emerging importance of oxidative stress in regulating striated muscle elasticity

**DOI:** 10.1007/s10974-014-9392-y

**Published:** 2014-11-06

**Authors:** Lisa Beckendorf, Wolfgang A. Linke

**Affiliations:** Department of Cardiovascular Physiology, Institute of Physiology, Ruhr University Bochum, MA 3/56, 44780 Bochum, Germany

**Keywords:** Oxidative modification, Myofilaments, Sarcomere proteins, Titin, Passive tension, Diastolic stiffness

## Abstract

The contractile function of striated muscle cells is altered by oxidative/nitrosative stress, which can be observed under physiological conditions but also in diseases like heart failure or muscular dystrophy. Oxidative stress causes oxidative modifications of myofilament proteins and can impair myocyte contractility. Recent evidence also suggests an important effect of oxidative stress on muscle elasticity and passive stiffness via modifications of the giant protein titin. In this review we provide a short overview of known oxidative modifications in thin and thick filament proteins and then discuss in more detail those oxidative stress-related modifications altering titin stiffness directly or indirectly. Direct modifications of titin include reversible disulfide bonding within the cardiac-specific N2-Bus domain, which increases titin stiffness, and reversible *S*-glutathionylation of cryptic cysteines in immunoglobulin-like domains, which only takes place after the domains have unfolded and which reduces titin stiffness in cardiac and skeletal muscle. Indirect effects of oxidative stress on titin can occur via reversible modifications of protein kinase signalling pathways (especially the NO-cGMP-PKG axis), which alter the phosphorylation level of certain disordered titin domains and thereby modulate titin stiffness. Oxidative stress also activates proteases such as matrix-metalloproteinase-2 and (indirectly via increasing the intracellular calcium level) calpain-1, both of which cleave titin to irreversibly reduce titin-based stiffness. Although some of these mechanisms require confirmation in the in vivo setting, there is evidence that oxidative stress-related modifications of titin are relevant in the context of biomarker design and represent potential targets for therapeutic intervention in some forms of muscle and heart disease.

## Introduction: Oxidative stress as an important modifier of myocyte properties

Oxidative stress occurs in the cell when reactive oxygen/nitrogen species (ROS/RNS) are increased or when the antioxidant defence mechanisms are decreased; i.e., when one or both of these factors go out of balance. Under pathological conditions, ROS can react with and thereby damage DNA, lipids and proteins, initiating tissue damage and cell death. However, at physiological concentrations, ROS can be critical regulators of cellular signalling pathways. ROS/RNS are increased, e.g., in myocardial ischemia/reperfusion (I/R) injury (Canton et al. 2004), in the course of heart failure (Haywood et al. 1996; Sawyer et al. 2002; Canton et al. 2011), and in various muscular dystrophies, such as dysferlinopathy (Terrill et al. 2013), Duchenne muscular dystrophy (DMD), and the mdx mouse model of DMD (Haycock et al. 1996; Disatnik et al. 1998; Kim et al. 2013; Canton et al. 2014). Among the targets of oxidative modification are various contractile and regulatory proteins of the sarcomeres, the structural and functional units of striated muscle. Oxidative modification of these myofilament proteins can have dramatic functional consequences, including altered calcium sensitivity of force production, contractile impairment and muscle weakness (Andrade et al. 2001; Smith and Reid 2006; Lamb and Westerblad 2011; Balogh et al. 2014), and sometimes also improvements in cardiac or skeletal muscle function (Gao et al. 2012; Lovelock et al. 2012; Mollica et al. 2012).

**Fig. 1:**
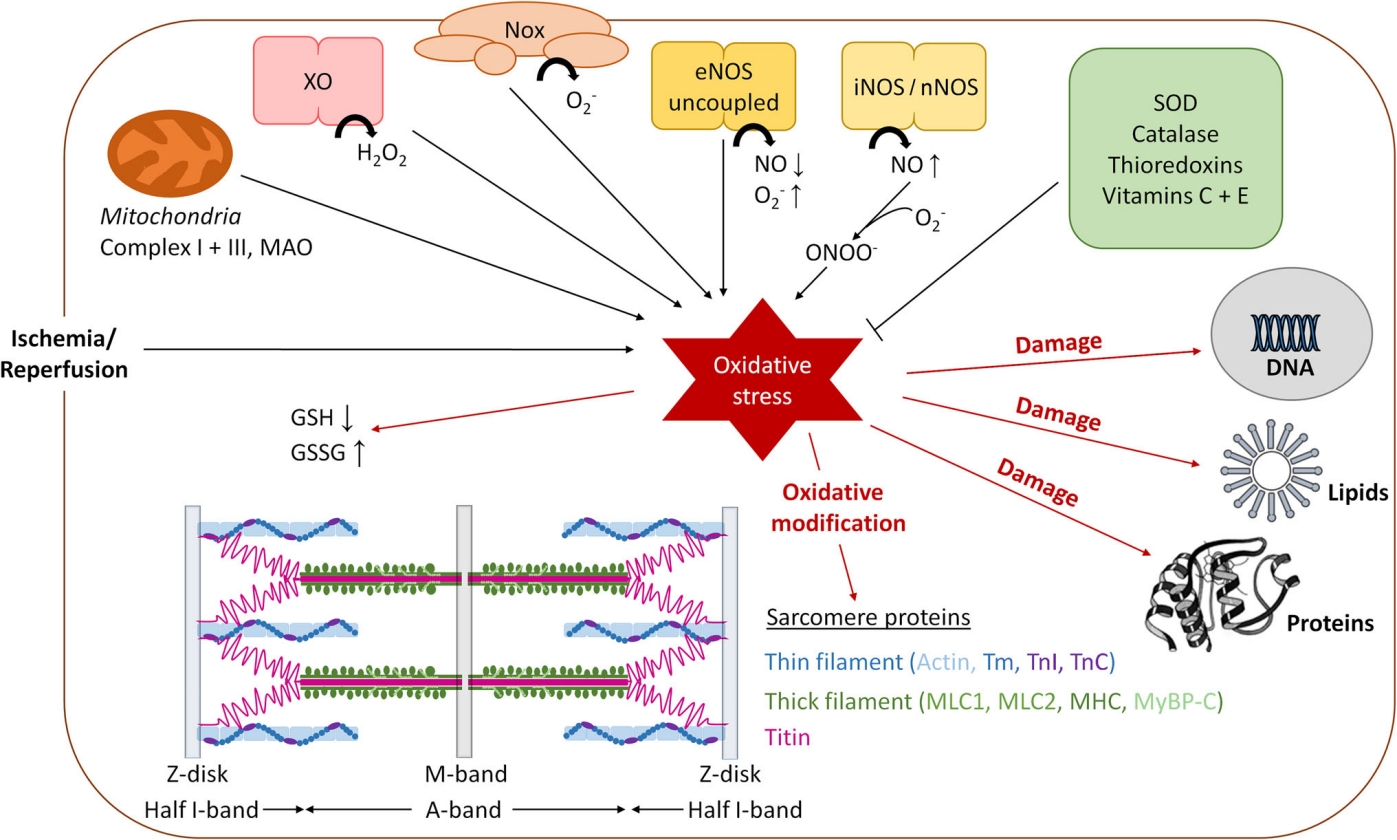
Schematic overview of important sources and targets of oxidative stress, as well as protectors against it, in striated muscle cells. Sources of reactive oxygen/nitrogen species include xanthine oxidase (XO), NADPH oxidases (Nox), uncoupled endothelial nitric oxide synthase (eNOS), inducible nitric oxide synthase (iNOS), neuronal nitric oxide synthase (nNOS), and mitochondrial factors such as complex I or III and monoamine oxidase (MAO). Antioxidant enzymes include superoxide dismutase (SOD), catalase, and thioredoxins, whereas non-enzymatic antioxidants are vitamins C and E. Oxidative stress damages DNA, lipids, and proteins, and among others, causes oxidation of myofilament proteins and alterations to the ratio between oxidized (GSSG) and reduced forms (GSH) of glutathione. Among the sarcomere proteins biochemically modified by oxidative stress are actin, tropomyosin (Tm), troponin I (TnI) and troponin C (TnC), myosin light chains 1 and 2 (MLC1 and MLC2), myosin heavy chain (MHC), myosin-binding protein-C (MyBP-C), and titin

Important sources of ROS in striated muscle cells (Fig. 1) include xanthine oxidase (XO) (Baldus et al. 2006), NADPH oxidases (Nox) (Heymes et al. 2003), uncoupled endothelial nitric oxide synthase (eNOS) (Xia et al. 1998), inducible nitric oxide synthase (iNOS) (Shah and MacCarthy 2000) as well as neuronal nitric oxide synthase (nNOS) (Zhang et al. 2014), and mitochondrial enzymes such as respiratory chain complex I or III and monoamine oxidase (MAO) (St-Pierre et al. 2002; Di Lisa et al. 2009). Well-known examples of ROS/RNS are hydrogen peroxide (H_2_O_2_), hydroxyl radicals (OH·), superoxide anions (O_2_^−^), and the highly reactive peroxynitrite (ONOO^−^), which is formed in the reaction of nitric oxide (NO) and O_2_^−^ (Fig. 1). Antioxidant defense mechanisms are also in place, involving enzymes such as catalase and superoxide dismutase (SOD), the thioredoxin system, as well as non-enzymatic factors like vitamins E and C (Fig. 1). ROS/RNS can alter miscellaneous cellular properties by reacting with amino acids in proteins. These proteins are then modified either reversibly (i.e., the oxidized protein can be enzymatically repaired) or irreversibly (i.e., the oxidized protein must be replaced by de novo synthesis), depending on the nature and amount of ROS (Canton et al. 2014). Reversible modifications caused by ROS/RNS include disulfide bridge formation, *S*-glutathionylation, nitrosylation, and sulfenylation; irreversible modifications include sulfinylation, sulfonylation, nitration, and carbonylation (Canton et al. 2014; Steinberg 2013). Frequent targets of oxidative modification are the thiol-containing amino acids, cysteine and methionine. Nitration affects predominantly tyrosine residues, whereas the main targets of carbonylation are lysine, arginine, threonine, and proline (Canton et al. 2014). Some of the modifications greatly impact protein structure and function, whereas for other modifications, the functional implications are incompletely understood or unknown. The reversibility of oxidative modifications can play a role in signal transduction processes and may also have a protective effect on the protein.

In this review we focus exclusively on the role of oxidative stress in altering the properties and functions of myofilament proteins. We begin with a brief overview of known oxidative modifications in thin and thick filament proteins, before discussing recent evidence for oxidative modifications of the giant titin filament, the protein responsible for the elasticity of cardiac and skeletal myocytes. We also touch on the pathophysiological implications of these findings and the potential for biomarker use and therapeutic intervention in disease. Overall, we make a case for the emerging importance of oxidative modifications of the titin springs in regulating myocyte elasticity and ‘passive’ stiffness under oxidative stress conditions.

## Impact of oxidative stress on thin filament proteins

Various myofilament proteins are biochemically and functionally altered under oxidative stress. Among these proteins are the components that constitute the sarcomeric thin filaments, actin, tropomyosin, and subunits of troponin (Fig. 1). Mass spectrometry identified actin among the *S*-thiolated cardiomyocyte proteins showing increased abundance in rat hearts following I/R (Eaton et al. 2002). Furthermore, *S*-glutathionylation of actin at Cys^374^ occurred already at baseline but was substantially elevated under ischemic conditions, and this oxidation impaired the interaction between actin and tropomyosin and the polymerisation of G-actin to F-actin (Dalle-Donne et al. 2003; Chen and Ogut 2006; Passarelli et al. 2010). *S*-glutathionylation of actin also reduced the activity of the actomyosin S1-ATPase (Pizarro and Ogut 2009). Additionally, carbonylation of actin caused disruption of the actin filaments in vitro (Dalle-Donne et al. 2001). Actin carbonylation was increased in end-stage failing human hearts and correlated with contractile impairment and reduced cardiomyocyte viability (Canton et al. 2011). Moreover, increased carbonylation of actin and other myofilament proteins was shown to be associated with a reduced Ca^2+^-sensitivity of force production in infarcted mouse hearts (Balogh et al. 2014).

Oxidation of the regulatory protein tropomyosin in microembolized pig hearts decreased contractile function, and this decrease correlated with the formation of tropomyosin homodimers (Canton et al. 2006). Tropomyosin dimer formation due to disulfide bonding was also detected in mouse cardiac tissue following myocardial infarction (Avner et al. 2012), in isolated rat hearts after postischemic reperfusion (Canton et al. 2004), and in failing rabbit hearts exposed to elevated oxidative stress caused by rapid left ventricular pacing (Heusch et al. 2010). Additionally, tropomyosin formed disulfide bridges with actin in H_2_O_2_-perfused rat hearts (Canton et al. 2004). Nitroxyl (HNO), a RNS activating signalling pathways different from NO (Miranda 2005), caused the formation of actin-tropomyosin heterodimers via actin Cys^257^ and tropomyosin Cys^190^ (Gao et al. 2012), which probably added to the beneficial effects on myocardial contractile function observed with HNO (Gao et al. 2012; Sabbah et al. 2013; Arcaro et al. 2014). In skeletal myocytes from the mdx mouse model of DMD, ROS production as well as the overall content of oxidized thiols were increased in comparison to wildtype animals, and tropomyosin cross-linking occurred (Menazza et al. 2010; El-Shafey et al. 2011). Nitration of tropomyosin was shown to occur in aging rat skeletal muscles (Kanski et al. 2005b).

The cardiac troponin subunits, cTnI and cTnC, contain tyrosine residues which are targets of nitration in aging rat hearts (Kanski et al. 2005a), although the functional impact from this biochemical modification is not known. The TnI isoform from fast-twitch skeletal muscle was identified as a target of *S*-glutathionylation in rat and human, and this modification increased the Ca^2+^ sensitivity of the contractile apparatus (Mollica et al. 2012). In this TnI isoform, Cys^133^ was the only accessible cysteine. Since the phosphorylation of a homologous serine in cTnI impedes the interaction with cTnC (Ward et al. 2001), oxidation of Cys^133^ in fast-twitch muscle TnI may also lead to a reduced binding affinity to TnC (Mollica et al. 2012).

Taken together, an established effect of oxidative modifications in thin filament proteins is the reduced myofilament Ca^2+^-sensitivity of force production (although this parameter can transiently increase under oxidative stress), which depresses contractile performance in both cardiac and skeletal muscle (Lamb and Westerblad 2011; Steinberg 2013). Oxidative stress-related effects on the structure of thin filament components and on the actin-myosin interface presumably contribute to the contractile impairment. In some cases, the contractile activity can be improved under oxidizing conditions (Steinberg 2013).

## Impact of oxidative stress on thick filament proteins

Thick filament proteins impaired by oxidative modifications include the myosin light chains 1 and 2 (MLC1 and MLC2), myosin heavy chain (MHC), and cardiac myosin-binding protein-C (cMyBP-C). As regards MLC1 and MLC2, tyrosine nitration (Tyr^73^ and Tyr^185^ in MLC1, and Tyr^182^ in MLC2) promoted the degradation of these proteins by matrix metalloproteinase-2 (MMP-2) (Doroszko et al. 2010; Polewicz et al. 2011). Nitrotyrosine-containing sequences from MLC were also detected in aging skeletal muscle (Kanski et al. 2005b). Oxidation of sulfhydryl groups in cysteines or methionines of MLC1 reduced the contractile force of human cardiomyocytes (Hertelendi et al. 2008).

MyBP-C appears to be modified by oxidative stress in various ways (Brennan et al. 2006). The protein showed similar levels of carbonylation in normal and infarcted mouse hearts (Balogh et al. 2014). Reversible *S*-glutathionylation of MyBP-C could be induced in detergent-extracted cardiac fibres in vitro by treatment with oxidized glutathione (GSSG) or reducing agent, dithiothreitol (DTT), and the sites of *S*-glutathionylation in MyBP-C were identified as Cys^479^, Cys^627^, and Cys^655^ (Patel et al. 2013). These oxidative modifications resulted in enhanced myofilament Ca^2+^ sensitivity and diastolic dysfunction (Lovelock et al. 2012; Patel et al. 2013).

MHC was found to be nitrated at several different tyrosine residues (Tyr^114^, Tyr^116^, Tyr^134^, and Tyr^142^) in aging rat heart (Hong et al. 2007) and increased MHC nitration negatively influenced the force generation of rat ventricular trabeculae (Mihm et al. 2003). Peroxynitrite-induced oxidation of two cysteines in MHC (Cys^697^ and Cys^707^) close to the catalytic centre inhibited the activity of the skeletal muscle S1-ATPase and reduced the maximum force (Tiago et al. 2006). Furthermore, in infarcted mouse hearts, the levels of MHC carbonylation were increased, which was suggested to partly explain the contractile impairment of these hearts (Balogh et al. 2014). Treatment of cardiomyocytes with HNO induced cross-bridge formation between cysteines of MHC and MLC1, and this modification was associated with an improved contractility (Gao et al. 2012). In conclusion, an increasing number of oxidative modifications are known to affect the major thick filament proteins, frequently with negative (but sometimes with positive) consequences for cardiomyocyte contractility. Oxidative modification can also predispose some thick filament proteins to increased degradation.

## Regulation of muscle elasticity via modifications of titin

**Fig. 2:**
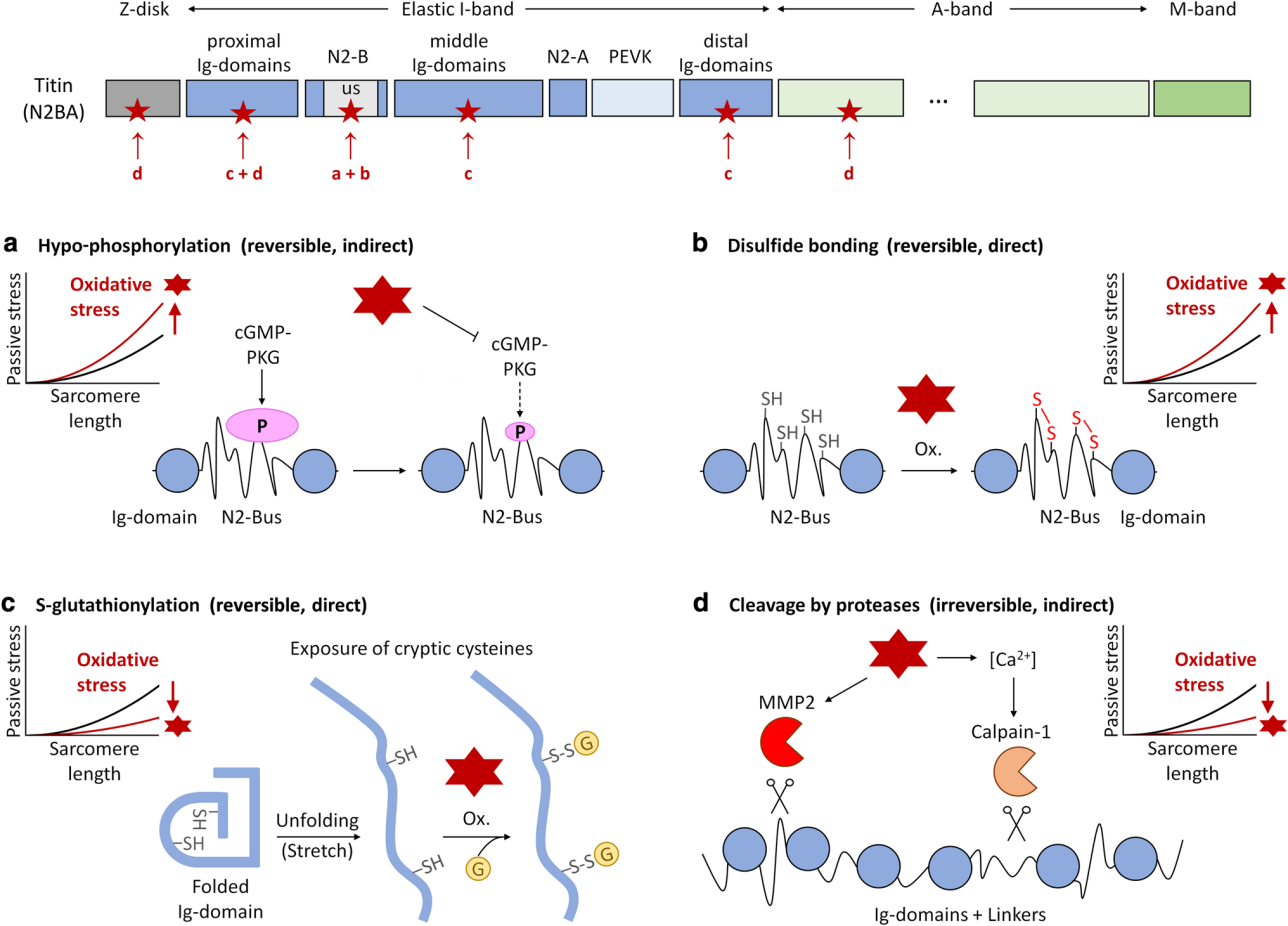
Oxidative stress-related modifications of titin affecting titin-based passive stiffness. The top panel illustrates the different segments of the titin chain (N2BA isoform) in a half-sarcomere, focusing on the various regions making up the elastic I-band segment. Segments where oxidative modifications occur are marked by arrows; the letters correspond to the respective type of oxidative modification indicated in panels (**a**–**d**). **a** Oxidative stress induces hypo-phosphorylation of the titin N2-Bus as it impairs NO-cGMP-PKG signalling; this modification increases titin stiffness. **b** Oxidizing conditions promote the formation of disulfide bonds in the titin N2-Bus; this modification increases titin stiffness. **c** Under oxidative conditions, buried cysteines in titin immunoglobulin (Ig-)domains are *S*-glutathionylated after they become exposed by domain unfolding (triggered by sarcomere stretch); this modification prevents domain refolding and thus reduces titin stiffness. **d** Oxidative stress increases the activity of proteases such as matrix metalloproteinase-2 (MMP2) and (via a rise in intracellular Ca^2+^ concentration) calpain-1, which degrade titin; these alterations would decrease titin stiffness

For the remainder of the review, we focus on the titin protein chain, the ‘third’ filament of the sarcomere next to the thin and thick filaments, and we begin with a brief discussion of some relevant titin properties (for a more comprehensive recent review, see Linke and Hamdani 2014). A well-established function of titin is to help determine the elastic properties of cardiac and skeletal muscles and to generate a ‘passive’ force upon stretching. The elasticity of titin resides within the extensible I-band portion of the protein, which is differentially spliced, giving rise to the major titin isoforms termed N2BA and N2B (both expressed in cardiac muscle) and N2A (expressed in skeletal muscle). I-band titin is composed of ‘proximal’, ‘middle’, and ‘distal’ (relative to the Z-disk) immunoglobulin-like (Ig-)domain regions; the PEVK domain rich in proline, glutamate, valine, and lysine, which is a disordered region; the N2-A element; and the cardiac-specific N2-B element, which contains a large disordered segment, the N2B-unique sequence (N2-Bus) (Fig. 2). The Ig-domain regions and the disordered segments are all involved in the molecular mechanism of titin elasticity (Linke 2000; Linke and Fernandez 2002; Li et al. 2002).

Titin stiffness is regulated in various different ways. In the long-term, the titin isoform size and variant can be altered (‘isoform switch’), which greatly affects myocyte passive stiffness. In the perinatal heart, a transition occurs from a highly compliant, fetal N2BA isoform (3.7 MDa) to shorter/less compliant N2BA isoforms and the short/stiff N2B titin (Lahmers et al. 2004; Opitz et al. 2004; Warren et al. 2004). This isoform transition can partially be reversed in the failing human heart, where the N2BA:N2B expression ratio increases again (Neagoe et al. 2002; Makarenko et al. 2004). In the short-term, titin stiffness is regulated by post-translational modifications (Linke and Hamdani 2014). Phosphorylation of the N2-Bus or the PEVK domain is mediated, e.g., by protein kinase (PK)A, cyclic guanosine monophosphate (cGMP) activated PKG, PKCα, or calcium/calmodulin-dependent protein kinase II (CaMKII), and these modifications—with the exception of the PKCα-mediated phosphorylation—decrease titin-based stiffness (Yamasaki et al. 2002; Krüger and Linke 2006; Krüger et al. 2009; Hidalgo et al. 2009; Hamdani et al. 2013c). In human heart failure, a phosphorylation deficit was observed, especially for PKG-mediated titin phosphorylation, and this was correlated with increased myocardial stiffness (Krüger et al. 2009; Kötter et al. 2013). Additional means by which titin stiffness can be modulated are now emerging, and these mechanisms are triggered by oxidative stress. The main purpose of this review is to discuss how ROS/RNS can modify the titin springs via different pathways, which can have opposing effects on the protein’s stiffness.

## Hypo-phosphorylation of titin due to impaired NO/cGMP/PKG signalling

NO produced by NOS enzymes (Fig. 1) activates soluble guanylyl cyclase (sGC) by binding to its heme moiety. The sGC then increases cGMP production and thereby activates PKG. This signalling mechanism is impaired by oxidative stress. Under oxidant conditions, eNOS becomes uncoupled by direct *S*-glutathionylation or via depletion of the enzyme’s co-factor, tetrahydrobiopterin, which decreases NO but increases the production of the highly reactive superoxide anion, O_2_^−^ (De Pascali et al. 2014). The lowered NO bioavailability reduces sGC activation and depresses the cGMP-PKG pathway. Moreover, the ferrous heme iron Fe^2+^ can be oxidized to Fe^3+^ under oxidative stress, further reducing the activity of sGC (Schrammel et al. 1996).

Due to the impaired NO/cGMP/PKG signalling under oxidizing conditions, titin may become hypo-phosphorylated mainly at the N2-Bus, which would increase the stiffness of the titin spring (Fig. 2a). Evidence that these alterations are presumably important in heart disease comes from the following observations: (i) a PKG-dependent titin phosphorylation deficit exists in failing human hearts, along with increased passive stiffness (Krüger et al. 2009; Kötter et al. 2013); (ii) a reduced myocardial cGMP concentration and PKG activity can be found in human and canine diastolic heart failure (van Heerebeek et al. 2012; Hamdani et al. 2013a); and (iii) increased nitrotyrosine levels are detectable in the hearts of diastolic heart failure patients (van Heerebeek et al. 2012). Furthermore, the pathologically high passive stiffness can be corrected ex vivo by administering cGMP-PKG to isolated cardiomyocytes (Borbély et al. 2009; van Heerebeek et al. 2012; Hamdani et al. 2013a; Hamdani et al. 2013b) and in vivo by boosting the cGMP-PKG pathway through pharmacological interventions in the dogs with diastolic heart failure (Bishu et al. 2011). These findings suggest that oxidative/nitrosative stress increases cardiac titin stiffness by impairing upstream signalling pathways relevant for PKG-mediated titin phosphorylation.

## Disulfide bridge formation in the cardiac titin N2-Bus

A direct oxidative stress-related modification of titin, which increases cardiomyocyte stiffness, is disulfide bonding in the cardiac-specific N2-Bus (Fig. 2b). Under oxidizing conditions, the six conserved cysteines present in the human N2-Bus can form up to three S–S bridges (Grützner et al. 2009). The disordered N2-Bus is thus mechanically stabilized and its extensibility is greatly impaired, as shown by single-molecule force-extension experiments on recombinant N2-B constructs using the atomic force microscope (AFM) (Grützner et al. 2009). Consistent with this, the reducing agent, thioredoxin, had a de-stiffening effect on isolated human cardiomyofibrils exposed to a cyclic stretch-release protocol (Grützner et al. 2009). Moreover, the maximum extension of the N2-Bus studied ex vivo by immunoelectron microscopy of stretched rabbit cardiac sarcomeres was only ~100 nm if a reducing agent was excluded from the medium (Linke et al. 1999), but ~200 nm if DTT (1 mM) was present (Trombitás et al. 1999). These values are very close to those measured for the N2-Bus in vitro using AFM force spectroscopy in the absence and presence of DTT, respectively (Grützner et al. 2009). Another aspect is that disulfide bonding in the N2-Bus most certainly also interferes with the regulation of titin stiffness by phosphorylation of this region. Indeed, it was observed that the de-stiffening effect of PKA on isolated cardiac myofibrils, which is caused by phosphorylation of the N2-Bus, is more pronounced in the presence of DTT than in the absence of it (Krüger and Linke 2006).

S–S bridge formation in titin’s N2-Bus may not only have a mechanical effect on the cardiomyocyte, but could also modify intracellular signalling pathways intersecting with the N2-Bus (Krüger and Linke 2011). This cardiac titin region binds the four-and-a-half LIM-domain proteins, FHL1 and FHL2 (Lange et al. 2002; Sheikh et al. 2008), and the small heat shock proteins (sHSPs), αB-crystallin and HSP27 (Bullard et al. 2004; Kötter et al. 2014). Disulfide bonds in the N2-Bus could alter these interactions and thus affect pathways of mechanosensation and protein quality control in the cardiomyocyte (Linke and Hamdani 2014). In conclusion, the N2-Bus of cardiac titin is a preferred target of oxidative modification in vitro and probably also in isolated cardiomyocytes. It remains to be established whether S–S bonding in the N2-Bus occurs under oxidative stress in vivo and if so, what impact this modification may have on myocardial stiffness and mechanical signalling.

## *S*-glutathionylation of cryptic cysteines in the Ig-domains of I-band titin

A recently elucidated direct modification of titin under oxidative stress is the *S*-glutathionylation of cryptic cysteines in the Ig-domains of the elastic I-band region (Alegre-Cebollada et al. 2014) (Fig. 2c). These cysteines are usually buried inside the Ig-domain fold but become exposed if the Ig-domain unfolds. Out of the maximally 93 Ig-domains present in the I-band titin spring, 89 domains contain cryptic cysteines that can potentially be oxidized upon domain unfolding. Interestingly, the I-band Ig-domains of titin contain, on average, between two and three cysteines, whereas most Ig-domains in all other parts of the titin molecule contain only one cysteine (Alegre-Cebollada et al. 2014). The majority of cysteines in the I-band Ig-domains are evolutionary well conserved. Some of these cysteines were suggested earlier to form disulfide bridges under oxidizing conditions, with the proximal and middle Ig-domains being a potential hotspot for such modifications (Mayans et al. 2001). However, single-molecule mechanical measurements by AFM force-clamp, using Ig-domain I91 (nomenclature of Bang et al. 2001), revealed that the two buried cysteines contained within this domain usually form mixed disulfides with glutathione in the presence of GSSG—but only if the domain is unfolded (Alegre-Cebollada et al. 2014) (Fig. 2c). The *S*-glutathionylation decreased the mechanical stability of the domain and prevented domain refolding. Importantly, to inhibit domain refolding, GSSG needed to be exposed for several tens of seconds, whereas exposure for only a few seconds had no or little effect. Treatment with reduced glutathione (GSH) or removal of the two cysteines by site-directed mutagenesis restored the ability of the Ig-domain to refold in the AFM experiments. Furthermore, *S*-glutathionylation of the unfolded I91 domain in the presence of GSSG was confirmed by Western blotting and was found to be fully reversible with the administration of DTT (Alegre-Cebollada et al. 2014). These findings showed for the first time that mechanical unfolding can enable oxidative modification of titin’s cryptic cysteines, which disrupt the domain folding/unfolding dynamics and cause sustained but reversible changes in titin elasticity.

Mechanical experiments on single skinned human cardiomyocytes demonstrated that oxidation by GSSG greatly reduces titin-based passive tension if the myocytes are exposed to the oxidizing agent in an over-stretched state favouring Ig-domain unfolding (Alegre-Cebollada et al. 2014). The reduction in cardiomyocyte stiffness is expected, because the unfolding of an Ig-domain causes a gain in contour length by ~30 nm compared to the folded state, such that the titin spring becomes longer and more extensible (Linke and Fernandez 2002). In the absence of over-stretch, GSSG still reduced cardiomyocyte stiffness by some amount (Alegre-Cebollada et al. 2014), due probably to a number of unfolded Ig-domains present in I-band titin at physiological sarcomere lengths (Linke and Fernandez 2002). The reduction in cardiomyocyte stiffness on GSSG-treatment was reversible with GSH or DTT.

In summary, evidence from in vitro and ex vivo experiments suggests that *S*-glutathionylation of cysteines in unfolded titin Ig-domains could be an important mechanism of myocyte stiffness regulation under oxidant stress, in both the heart and the skeletal muscles. In support of this notion, increased *S*-glutathionylation of sarcomere proteins was found in mouse heart tissue following myocardial infarction, and among these proteins was titin (Avner et al. 2012; Alegre-Cebollada et al. 2014). Future studies should explore how important this oxidative modification of titin is in the context of heart failure or muscle disease and to what degree it affects titin-based passive stiffness in vivo.

## Titin degradation by oxidative/nitrosative stress-activated proteases

Yet another way by which oxidative/nitrosative stress could alter titin stiffness is indirectly via activation of proteases that degrade titin (Fig. 2d). One of these proteases is MMP2, which is abundant in the cardiomyocyte (Kandasamy et al. 2010) and localizes to various subcellular compartments, including the Z-disk (Ali et al. 2010). MMP2 cleaved cardiac titin in a concentration-dependent manner and in rat hearts the titin cleavage was increased after myocardial I/R injury causing rapid induction of the highly pro-oxidant ONOO^−^ (Ali et al. 2010). Conversely, titin degradation induced by I/R damage was diminished by an MMP inhibitor. Previously, oxidative stress-activated MMP2 was shown to degrade various sarcomeric targets next to titin, including TnI, MLC1, and α-actinin (Wang et al. 2002; Sawicki et al. 2005; Sung et al. 2007). The MMP2-mediated structural alterations of sarcomeric proteins may be one reason for the reduced myocardial systolic and diastolic dysfunction observed with I/R injury (Linke 2010).

The Ca^2+^-dependent intracellular protease, calpain-1, also degrades titin in cardiomyocytes, preferentially within the elastic spring segment, and calpain inhibitors prevent this degradation (Lim et al. 2004; Barta et al. 2005). Although there is no evidence for direct activation of calpain-1 by oxidant stress, the protease is thought to be induced by cardiac I/R damage due to Ca^2+^ overload (Inserte et al. 2012). This increase in calcium levels through oxidative stress occurs by various means, especially via activation/sensitization of the ryanodine receptor Ca^2+^-release channels (Allen et al. 2008). Interestingly, in the presence of Ca^2+^, calpain-1 binds to titin’s Ig-domain I4 in the proximal I-band region (titin domain nomenclature of Bang et al. 2001) where it could be “stored until further use” in the myocyte (Coulis et al. 2008). A remarkable observation in this context is that titin is more susceptible to calpain-1-mediated proteolysis when it is stretched (Murphy et al. 2006), suggesting that in extended, or perhaps overstretched, sarcomeres titin is particularly susceptible to such proteolysis. Taken together, current evidence suggests that preferential proteolysis of I-band titin by activation of calpain-1 is an early process in myocyte injury and that oxidative stress may play a role in this structural damage.

Proteolytic degradation of the titin spring segment induced by oxidative stress will decrease the passive stiffness of the myocytes irreversibly (Fig. 2d). Active contraction will also be compromised, as the damage to I-band titin impairs the accurate positioning of the thick filaments in the middle of the sarcomere and thus, force generation by actomyosin (Horowits et al. 1986). Moreover, titin is important for the length-dependent activation of cardiac and skeletal myocytes (Fukuda and Granzier 2005; Mateja et al. 2013) and titin proteolytic damage will depress this function. Increased oxidative stress and severe titin degradation can be observed in human ischemic cardiomyopathy (Hein et al. 1994; Morano et al. 1994), suggesting that a connection exists between these two events, although a causative relationship remains to be proven.

## Considerations on the possible net effect of oxidative titin modifications on cell stiffness

The various direct and indirect effects of oxidative stress on titin (Fig. 2) may occur concomitantly with one another, which would make it unpredictable in which direction they alter the stiffness of the myocyte. Whereas the titin phosphorylation deficit and the disulfide bonding in the N2-Bus will increase titin-based stiffness, the *S*-glutathionylation of cryptic cysteines and the irreversible protease-dependent titin cleavage will decrease it. Which one of these effects may be dominating under which physiological or disease condition in the heart or the skeletal muscles remains to be seen. Notably, the oxidative modifications directed at the titin N2-Bus (Fig. 2a, b) can occur in cardiac but not in skeletal myocytes, because only the former express the N2-Bus-containing titin isoforms (N2BA, N2B). In contrast, the protease-mediated titin degradation and the *S*-glutathionylation of cryptic cysteines in titin Ig-domains can take place in both cardiac and skeletal muscle. This *S*-glutathionylation presumably requires increased muscle stretch (increased cardiac preload) in order to exert a significant effect on (cardio) myocyte stiffness. Thus, the higher the preload on the cardiac chamber filled under oxidative stress, the more pronounced may be the mechanical weakening due to oxidized, unfolded titin Ig-domains. Along the same line, pre-stretch of a skeletal muscle to long sarcomere length under oxidant conditions may have a noticeable softening effect on that muscle. One can also speculate that oxidative stress in conjunction with high stretch could have a de-stiffening effect on skeletal myocytes but not on cardiomyocytes, because in the latter the different means of oxidative titin modifications may neutralize one another in their effect on total passive stiffness.

Oxidative stress is often coupled with other important changes to the intracellular milieu, especially acidosis (e.g., during I/R). Both these conditions evoke a protective response by the myocyte mediated by inducible heat shock proteins, such as the sHSPs, αB-crystallin and HSP27 (Mymrikov et al. 2011; Larkins et al. 2012). Under oxidant/acidic stress, these chaperones associate preferentially with the I-band titin springs in both cardiac and skeletal myocytes (Bullard et al. 2004; Kötter et al. 2014). Importantly, the titin-sHSP interaction affects titin stiffness. Folded titin Ig-domains appear to be stabilized mechanically by this interaction (Bullard et al. 2004), whereas unfolded Ig-domains are protected from aggregation by sHSP-binding, which prevents excessive myocyte stiffening (Kötter et al. 2014). Whether this binding of sHSPs would interfere with the exposure of cryptic cysteines and their *S*-glutathionylation under oxidizing conditions is unknown. However, the sHSP-titin binding adds to the complexity of possible effects of oxidative stress on titin-based stiffness.

Last but not least, oxidative modifications have been shown to increase the activity of several protein kinases, including PKA, PKG, PKC, and CaMKII (reviewed by Steinberg 2013), and to reduce the activity of protein phosphatases (Wright et al. 2009). Since the phosphorylation state of I-band titin affects titin-based stiffness (Linke and Hamdani 2014), any oxidative stress-mediated increase in kinase activity or reduction in phosphatase activity will also have an impact on myocyte stiffness. In conclusion, while oxidative stress seems almost certain to alter titin stiffness via multiple mechanisms in vivo, the magnitude and the direction of the stiffness modulation need to be established in additional studies.

## Oxidative titin modification as a potential biomarker and therapeutic target

Since oxidative stress plays a crucial role in the pathology of various cardiac and skeletal muscle diseases (see Introduction), the question arises whether oxidative modifications in titin may be characteristic of some of those conditions. Interestingly, in Chagas’ disease, which is caused by *Trypanosoma cruzi* infection but presents with severe cardiac symptoms (cardiomegaly, ventricular dilatation), the increased oxidative/nitrosative stress associated with this disease was shown to cause nitration of Ig-repeats from the cardiac N2B-titin isoform, and the nitrated peptides were detectable in the plasma from a rat model and from patients (Dihman et al. 2008). The nitrated titin was also recognized by antibodies from the host’s immune system and evoked a self-directed immune response (Dihman et al. 2012). Thus, ROS/RNS-dependent modifications of titin could indeed serve as biomarkers of specific forms of cardiac and skeletal muscle disease. In this context, titin has recently been suggested as a specific biomarker of DMD detectable in urine samples of affected patients and in serum samples from the mdx mouse (Rouillon et al. 2014; Hathout et al. 2014). Since oxidative stress is an established hallmark of this muscle disease, it may be worth extending the analysis to oxidated/nitrated titin peptide species to improve marker specificity.

Oxidative titin modifications could also serve as potential therapeutic targets in skeletal or heart muscle diseases associated with myocyte stiffening. While cardiomyocyte stiffening is well-documented especially in diastolic heart failure (Linke and Hamdani, 2014), skeletal muscle fibres can also get stiffer under disease conditions, e.g., in certain neurological disorders (Olsson et al. 2006; Mathewson et al. 2014). An interesting treatment option in heart failure associated with elevated diastolic stiffness may arise from the fact that oxidative stress modulates the NO-cGMP-PKG pathway, an important modifier of titin-based stiffness. In the transition to heart failure, oxidative stress can be triggered by co-morbidities, such as old age, renal insufficiency, obesity, diabetes mellitus, or hypertension, all of which can increase ROS/RNS levels (Paulus and Tschöpe 2013). Oxidative stress would reduce NO bioavailability, block sGC activity, down-regulate cGMP-PKG signalling, and thus cause hypo-phosphorylation of titin at the N2-Bus and pathologically increased passive tension. A (diastolic) heart failure patient may well benefit from the use of NO donors, inhibitors of cGMP-degrading enzymes, antioxidants, or other drugs that block the oxidative-stress effects on titin stiffness (Gladden et al. 2014), in that cardiomyocyte stiffness will be reduced and myocardial diastolic function improved.

Finally, a yet speculative opportunity to help improve symptoms in some cardiac (and skeletal myopathy?) patients may involve promoting the oxidative/nitrosative modification of cysteines in unfolded titin Ig-domains. For instance, when treating patients or dogs in acute heart failure with HNO donors (e.g., Angeli’s salt), improvements in both systolic and diastolic mechanical properties (including diastolic stiffness) were observed (Sabbah et al. 2013; Arcaro et al. 2014). The de-stiffening effect in diastole could be due in part to a reduced titin stiffness resulting from nitrosative modification (*S* **-**nitrosylation) of cysteines in I-band titin Ig-domains, similar to the effect of *S*-glutathionylation on these domains (Alegre-Cebollada et al. 2014). Notably, the HNO donors are considered to exert their effects independent from cGMP-PKG (and cAMP-PKA) signalling.

In conclusion, recent evidence suggests that oxidative/nitrosative stress-related modifications of titin occur in both cardiac and skeletal myocytes. These modifications can alter titin-based passive stiffness and perhaps modulate additional functions of titin. To which degree the oxidative modifications of the titin springs may be relevant for myocyte stiffness in striated muscle disease, remains to be seen. However, oxidative changes in titin have the potential to serve as biomarkers and become useful drug targets in specific forms of muscle/heart disease.
